# Prevalence and Factors Related to Natural Resistance-Associated Substitutions to Direct-Acting Antivirals in Patients with Genotype 1 Hepatitis C Virus Infection

**DOI:** 10.3390/v11010003

**Published:** 2018-12-21

**Authors:** Isabella Esposito, Sebastián Marciano, Leila Haddad, Omar Galdame, Alejandra Franco, Adrián Gadano, Diego Flichman, Julieta Trinks

**Affiliations:** 1 Instituto de Medicina Traslacional e Ingeniería Biomédica (IMTIB), CONICET, Instituto Universitario del Hospital Italiano (IUHI), Hospital Italiano (HIBA), C1199ACL Buenos Aires, Argentina; isabellesposito@gmail.com (I.E.); alejandra.franco@hospitalitaliano.org.ar (A.F.); adrian.gadano@hospitalitaliano.org.ar (A.G.); 2 Sección de Hepatología, Servicio de Clínica Médica, Hospital Italiano de Buenos Aires, C1199ABB Buenos Aires, Argentina; sebastian.marciano@hospitalitaliano.org.ar (S.M.); leila.haddad@hospitalitaliano.org.ar (L.H.); omar.galdame@hospitalitaliano.org.ar (O.G.); 3 Departamento de Investigación, Hospital Italiano de Buenos Aires, C1199ABB Buenos Aires, Argentina; 4 Cátedra de Virología, Facultad de Farmacia y Bioquímica, Universidad de Buenos Aires, C1113AAD Buenos Aires, Argentina; dflichman@ffyb.uba.ar; 5 Consejo Nacional de Investigaciones Científicas y Técnicas (CONICET), C1425FQB Buenos Aires, Argentina

**Keywords:** hepatitis C virus, resistance-associated substitutions, direct-acting antivirals, quasispecies

## Abstract

This study aimed to assess the prevalence of natural resistance-associated substitutions (RASs) to NS3, NS5A and NS5B inhibitors in 86 genotype 1 Hepatitis C Virus (HCV)-infected patients from Buenos Aires, Argentina, and to determine their effect on therapy outcome. Additionally, virological, clinical and host genetic factors were explored as predictors of the presence of baseline RASs. *NS3* RASs (39.2%) were more prevalent than *NS5A* RASs (25%) and *NS5B* RASs (8.9%). In the three regions, the frequencies of RASs were significantly higher in HCV-1b than in HCV-1a. The prevalence of Y93H, L159F and Q80K were 1.3%, 6.3% and 2.5%, respectively. IFNL3 CC genotype was identified as an independent predictor of the presence of baseline RASs in *NS5A* and *NS3* genes (*p* = 0.0005 and *p* = 0.01, respectively). Sustained virologic response was achieved by 93.3% of the patients after receiving direct-acting antivirals (DAAs), although 48.7% of them showed baseline RASs related to the DAA-regimen. Notably, the prevalence of clinically relevant RASs in the three genes was lower than that observed around the world. The baseline presence of RASs in both subtypes did not appear to affect therapy outcome. These results support the need to evaluate resistance patterns in each particular country since RASs´ prevalence significantly vary worldwide.

## 1. Introduction

Hepatitis C Virus (HCV) infection affects more than 70 million people worldwide [1,2]. Argentina is considered a low-endemicity country, with a prevalence that is estimated to be around 1% [3]; although higher rates have been described in different small rural communities [4].

The treatment of chronic HCV infection has considerably improved in recent years with the widespread development of “direct-acting antiviral” (DAA) drugs. These drugs, which target specific viral proteins of the HCV lifecycle, can be combined in highly effective, well-tolerated, interferon-free treatment regimens [5,6]. Currently available HCV DAAs are classified into four categories on the basis of their molecular target in the viral replication cycle and mechanism of action: NS3/4A protease inhibitors, NS5A inhibitors, nucleotide analogue inhibitors of NS5B RNA-dependent RNA polymerase (RdRp), and non-nucleoside inhibitors of RdRp. Each drug family has a specific resistance profile that influences the barrier to resistance and may vary among HCV genotypes and subtypes [7,8]. In this regard, different combinations of simeprevir (SMV), sofosbuvir (SOF), paritaprevir (PTV), daclatasvir (DCV), ledipasvir (LDV), ombitasvir (OMV), dasabuvir (DSV), velpatasvir (VLP), grazoprevir (GZR) and elbasvir (ELB), have already been approved by the regulatory system and are currently available in Argentina [9].

Despite the high rates of sustained virologic response (SVR) (over 90%), the effectiveness of new DAAs may be compromised by the emergence of resistance-associated variants (RAVs) [6,10,11,12]. The development of drug resistance is indeed an intrinsic, and to some extent unavoidable, characteristic of antiviral therapies. The combination of the high replication rate of HCV, along with the low fidelity and the lack of proof-reading mechanisms of its RNA dependent RNA polymerase, results in a highly variable viral population known as “quasispecies” within each infected individual. This large heterogeneity in viral gene sequences and natural dynamic variability promotes the rapid emergence of RAVs [13].

An RAV is defined by the presence of one or more resistance-associated substitutions (RASs), amino acid substitutions able to adversely impact the activity of DAAs in vitro and/or in vivo in treated patients [14]. Resistance-associated substitutions (RASs) may arise during therapy or may pre-exist at baseline, increasing the likelihood of treatment failure [6,10,11,12,15]. In particular, natural changes in HCV *NS3*, *NS5A* and *NS5B* genes associated with reduced drug sensitivity have been observed in DAA treatment-naïve patients [6]. Therefore, even prior to treatment, RAVs may exist as minor variants at baseline, which would rapidly become dominant under the selective pressure exerted by the drugs, subsequently leading to a virological breakthrough during treatment or a relapse after treatment cessation [6,14].

The prevalence of these naturally occurring RASs has been examined using standard population (Sanger) sequencing. Unfortunately, this conventional method is not sensitive enough in detecting clinically relevant variants present in less than 20% of the viral population [16]. In this regard, next-generation sequencing (NGS) technologies have demonstrated to be a useful tool to detect minor variants at baseline [17].

The utility of RAS testing depends upon both patient characteristics and DAA regimen. At present, RASs detection at baseline is particularly important in patients infected with HCV genotypes 1a and 3 [12]. Even though treatment-associated RASs are clinically more important than natural RASs, the latter might negatively impact treatment with some regimens like ELB/GZR and SMV/SOF in patients infected with genotype 1a [12]. Nevertheless, until newer DAAs become extensively available in all countries, and the issue of resistance will not be overcome, the HCV genotypic resistance testing is, and will be, an essential diagnostic tool for tailoring personalized treatments, particularly after a DAA-failure [12].

Emerging data have suggested that complex interactions between factors related to the infecting virus (genotypes, viral load, RASs) and to the host (age, gender, degree of liver fibrosis, alcohol consumption, etc.) would predict HCV treatment success and/or improve safety [8,18]. In fact, significant associations have been reported between natural RASs and host genetic determinants in the interferon lambda 3 (IFNL3) and 4 (IFNL4) genes, identified as predictors of Pegylated Interferon and Ribavirin (PegIFN/RBV) response in chronic HCV [19,20,21].

Given that natural RASs that might confer DAAs resistance exhibit geographical differences in their frequencies [22], the interpretation of the resistance profile is very complex, and the need of resistance testing should be defined in each country. In this regard, the prevalence of natural RASs has not been extensively studied in Argentina. Therefore, the aim of this study was to estimate the prevalence of RASs within *NS3*, *NS5A* and *NS5B* genomic regions in DAA-naïve patients chronically infected with HCV genotype 1, by automated Sanger sequencing and Ion Torrent NGS, and to determine their effect on therapy outcome. Additionally, virological, clinical and host genetic factors were explored as predictors of the presence of baseline RASs. 

## 2. Materials and Methods 

### 2.1. Study Population

This study was approved a priori by the Ethics Committee on Research from the Hospital Italiano of Buenos Aires and conducted in accordance with good clinical practice guidelines and the principles of the Declaration of Helsinki.

From 2012 to 2014, consecutive DAA-naïve patients with genotype 1 chronic hepatitis C were invited to participate in the study, which took place at the Hepatology Unit of the Hospital Italiano of Buenos Aires. Serum and whole blood samples were collected from each patient, after obtaining written informed consent.

Clinical data, such as gender, age and previous failure to PegIFN/RBV treatment, were recorded. To evaluate the impact of baseline RASs on treatment outcome, SVR rates were documented in those patients who underwent DAA prescription after recruitment and sample collection. Fibrosis grade was staged either by biopsy or Transient Elastography by Fibroscan® (Echosens, Paris, France). Plasma HCV RNA load was measured using Cobas® TaqMan® (Roche, Pleasanton, CA, USA), with a detection limit of 15 IU/mL. HIV co-infection was diagnosed by ELISA (Dade Behring; Enzygnost anti HIV-1/2 plus, Marburg GmbH, Germany) and confirmed by Western-blot (New Lab Blot-1, Bio-Rad, Marnes-la-Coquette, France).

### 2.2. RT-PCR and Automated Sanger Sequencing

*NS3*, *NS5A* and *NS5B* genomic regions were partially amplified by previously described RT-Nested PCR protocols specific for subtype 1a and 1b [23,24,25], covering positions involved in drug resistance. PCR products were bi-directionally sequenced using the Big-Dye Termination chemistry system (Applied Biosystems, Foster City, CA, USA). 

HCV genotype and subtype were confirmed in each genomic region by phylogenetic analysis. BioEdit (v.7.2.5) software [26] was used for sequence alignment. Phylogenetic trees were constructed using the maximum-likelihood method in MEGA (v.6.0) [27], and visualized in TreeView v.1.6.6 [28]. Nucleotide sequences were deposited in GenBank under accession numbers MH733012–MH733240 and MK215006–MK215017.

### 2.3. NGS Sequencing 

Amplicons were fragmented into 200 base pair (bp) pieces using Bioruptor® Sonication System (Diagenode, Denville, NJ, USA). Fragments were nick repaired, adaptor ligated, and barcoded using the Ion Neb Next Library kit (Life Technologies, Foster, CA, USA). Then, fragments were purified by Agencourt® AMPure® XP magnetic beads (Beckman Coulter, Brea, CA, USA), the fragmentation pattern was analyzed by semi-fluidic electrophoresis (Agilent® Bioanalyzer®, Santa Clara, CA, USA) and libraries were diluted to obtain equimolar amounts. Emulsion PCR was carried out using the Ion OneTouchTM 2.0 system (Life Technologies, Carlsbad, CA, USA) and the Ion PGMTM Template Hi-Q™ OT2 200 kit (Thermo Fisher Scientific, Waltham, MA, USA), followed by an enrichment step. Sequencing was performed on an Ion Torrent Personal Genome Machine (PGM) sequencer (Thermo Fisher Scientific, Waltham, MA, USA) using the Ion PGM™ Hi-Q™ Sequencing Kit and the Ion 316 chip (Thermo Fisher Scientific, Waltham, MA, USA).

### 2.4. RASs Analysis 

RASs were defined as a change in the RNA sequence associated to in vivo (with treatment failure) and/or in vitro phenotypic assays (to confer more than 2.5-fold change in drug susceptibility in comparison to the wild-type reference strain). RASs summarized by a recent systematic review [15] were selected for the analysis in this study. Overall, nine NS5A (24, 28, 30, 31, 32, 38, 58, 92 and 93), three NS5B (159, 237 and 282) and 14 NS3 positions (36, 41, 43, 54, 55, 56, 80, 122, 155, 156, 158, 168, 170 and 175) were analyzed in this study. Nucleotide sequences obtained from automated Sanger sequencing were aligned and compared with reference sequences for subtype 1a (AF009606) and 1b (AJ238799), using BioEdit (v.7.2.5) software [26].

All NGS reads were trimmed and aligned with the previously mentioned reference sequences using the Smith–Waterman algorithm, included in the Torrent Suite™ software (Thermo Fisher Scientific, Waltham, MA, USA). The plugins Variant Caller and Coverage Analyzer, both available in this software, were used for analysis of sequence variants and coverage, respectively. Low stringency parameters were used to identify variants, as previously reported [17]. Sequence data were visually confirmed with the Integrative Genomics Viewer and any sequence, alignment, or variant call error artifacts were discarded.

### 2.5. HCV Quasispecies Analysis

Sequencing errors induced by PCR and ultra-deep sequencing would complicate the analysis of viral populations and result in inflated estimates of quasispecies heterogeneity. To avoid such misinterpretation, all necessary precautions in sample preparation, RNA extraction, and amplification were taken into account [29], and PCR bias was avoided as previously reported [30]. In addition, ShoRAH (v.0.8) software, which applied a probabilistic Bayesian approach to minimize the effects of errors, was used [31].

The quasispecies complexity of *NS3*, *NS5A* and *NS5B* was determined by calculating: Nucleotide mutation frequency (Pn) and normalized Shannon entropy (Sn). Pn was calculated as the total number of polymorphic sites divided by the total number of nucleotides sequenced; considering that the variability of the quasispecies increased as Pn increased. The measure of the Shannon entropy, defined in terms of the probabilities of different sequences that can appear at a given time point was calculated using the formula S = −Σi(pi ln pi), where pi is the frequency of each variant in the viral quasispecies. The normalized entropy, Sn, was calculated as Sn = S/ln N, where N is the total number of sequences analyzed in each sample. Sn theoretically varies from 0 (no diversity) to 1 (maximum diversity) [32].

### 2.6. SNPs Genotyping

SNPs rs12979860 (IFNL3) and rs368234815 (IFNL4) were genotyped by PCR [21] followed by bi-directional sequencing using Big-Dye Termination chemistry system. BioEdit (v.7.2.5) software [26] was used to discriminate between homozygotes and heterozygotes.

### 2.7. Evaluated Variables and Statistical Analyses

For descriptive statistics, mean and standard deviation (SD) or absolute number and percentages were used. The statistical analysis of data was performed by means of contingency tables using Fisher exact probability test for categorical variables and Mann–Whitney U test for continuous variables. Univariate and multivariate logistic analyses were performed to identify variables that were independently associated with the presence of RASs in each genomic region. All significant parameters in the univariate analysis were included in the multivariate analysis. The virological variables that were explored to be associated with the presence of RASs were HCV subtypes, viral load and quasispecies complexity. Age, gender, METAVIR score and previous failure to PegIFN/RBV treatment were the clinical variables analyzed to be associated with RASs. Host SNPs in IFNL3/IFNL4 genes were also explored. Statistical analyses were performed with the Statistical Package for the Social Sciences software (v.18.0) (SPSS, Chicago, IL, USA) and significant differences were considered only for *p* < 0.05.

## 3. Results

### 3.1. Patients’ Characteristics

A total of 86 consecutive DAA-naïve patients chronically infected with HCV genotype 1 (33 subtype 1a and 53 subtype 1b) were included in the study. The clinical, virological and host genetic characteristics of the patients are shown in Table 1.

### 3.2. Prevalence of RASs in NS5A Protein

The *NS5A* region was successfully amplified in 84 (97.7%) samples: 51 (60.7%) belonged to HCV subtype 1b and 33 (39.3%) to subtype 1a. The latter sequences clustered into clade 1 (25 sequences; 75.8%) and clade 2 (eight sequences; 24.2%) (Supplementary Figure S1). No difference was observed in the presence of RASs between both clades: 8/25 (32%) in clade 1 and 4/8 (50%) in clade 2 (*p* = 0.42).

A total of 14 (16.7%) sequences exhibited RASs among the 84 analyzed patients, being the prevalence significantly higher in subtype 1b (13/51; 25.5%) when compared to subtype 1a (1/33; 3%) (*p* = 0.007).

Among the 51 subtype 1b sequences, RASs L28M (2%), L31M (2%), L31F (2%), and R30Q (13.7%) considered as primary resistance mutations to DCV, ELB, LDV and OMV, were identified. Substitutions detected at positions 58—which confers resistance to VLP—and 92—associated with resistance to LDV and OMV—were P58R/T (4%) and A92T (2%), respectively (Table 2).

Among the 33 subtype 1a strains, the primary resistance mutation M28V, related to resistance to LDV and OMV, was observed in one (3%) sample. (Table 2).

No combinations of multiple RAS were observed in subtype 1a, whereas in subtype 1b, one patient (2%) carried two mutations being the genetic profile R30Q+P58R.

### 3.3. Prevalence of RASs in NS5B Polymerase

The *NS5B* region was successfully amplified in 79 (91.9%) samples: 49 (62%) belonged to HCV subtype 1b and 30 (38%) to subtype 1a. The latter sequences split into clade 1 (22 sequences; 73.3%) and clade 2 (eight sequences; 26.7%) (Supplementary Figure S2). No difference was observed in the presence of RASs between both clades: 4/22 (18.2%) in clade 1 and 3/8 (37.5%) in clade 2 (*p* = 0.34).

Screening and analysis of NS5B residues 1 to 300 detected RASs at positions 159 and 282 in seven (8.9%) of the 79 sequences, all of them ascribed to subtype 1b. Therefore, the prevalence of RASs was significantly higher in subtype 1b (7/49; 14.3%) when compared to subtype 1a (0%) (*p* = 0.04). 

Among the 49 subtype 1b sequences, five (10.2%) harbored the L159F variant, associated with a lower response to SOF. Although the major mutation S282T, which confers high-level resistance to SOF, was not observed, RASs S282G (4.1%) was detected (Table 3). 

No combinations of multiple RASs were observed in subtype 1a- or 1b-infected patients.

### 3.4. Prevalence of RASs in NS3 Protease

The *NS3* gene was successfully amplified in 79 (91.9%) samples: 50 were ascribed to HCV subtype 1b (63.3%) and 29 to subtype 1a (36.7%). The latter sequences clustered into clade 1 (22 sequences; 75.9%) and clade 2 (seven sequences; 24.1%) (Supplementary Figure S3). No difference was observed in the presence of RASs between both clades: 5/22 (22.7%) in clade 1 and 3/7 (42.9%) in clade 2 (*p* = 0.36).

RASs were found in 22 (27.8%) of the 79 sequences, and their overall prevalence was higher in subtype 1b (18/50; 36%) than in subtype 1a (4/29; 13.8%) (*p* = 0.04).

Among the 29 subtype 1a strains, the clinically relevant RASs Q80K, associated with resistance to SMV, GZR and PTV, was detected only in one (3.4%) sample ascribed to subtype 1a clade 1. Substitution T54S, associated with resistance to ASV and SMV, was found in one (2%) sequence; as well as RAS V36L, which confers resistance to SMV, GZR and PTV, and I170V associated with resistance to SMV (Table 4).

Among the 50 subtype 1b strains, eight (16%) sequences presented the mutation Y56F associated with resistance to GRZ, and six (12%) exhibited the substitutions S122G/T associated with resistance to SMV. Variants at position 80 were detected in two (4%) samples: The primary resistance RAS Q80K and the low-level resistance RAS to SMV Q80R. Substitution T54S which confers resistance to first-generation protease inhibitors, and R155 associated with resistance to first- and second-generation protease inhibitors, were found each in one (2%) sequence (Table 4).

No combinations of multiple RASs were observed in subtype 1a- or 1b-infected patients.

### 3.5. Comparison of NGS and Direct Sequencing Results 

The *NS5A*, *NS5B* and *NS3* genes were successfully sequenced by NGS in 76 (90.5%), 72 (91.1%) and 72 (91.1%) of the samples characterized by automated Sanger sequencing, respectively. A mean of 12,259,169, 10,968,036 and 19,235,648 bp in the *NS5A*, *NS5B* and *NS3* genes were mapped onto the reference sequences, and an overall average coverage depth of 889x, 859x and 987x was achieved for each region, respectively. An average of 60,676, 59,412 and 90,748 reads of the *NS5A*, *NS5B* and *NS3* regions were aligned and compared with reference sequences for each subtype, respectively. Deep sequencing yielded a median of 11,650, 10,870 and 12,340 sequences per sample in the *NS5A*, *NS5B* and *NS3* genomic regions, respectively.

All RASs called by automated Sanger sequencing were confirmed by the NGS platform. Furthermore, deep sequencing (interpretative cut-off of ≥1%) identified additional RASs with frequencies between 2% and 21% among the patient’s viral quasispecies, in *NS5A* (8/76; 10.5%) and *NS3* (9/72; 11.1%) but not in *NS5B* (Table 5).

### 3.6. Assessment of HCV Quasispecies Diversity

The viral population parameters of complexity of the *NS5A*, *NS5B* and *NS3* genes sequenced by NGS, are summarized in Table 6.

No significant associations were observed when these parameters were compared among the three analyzed HCV genomic regions. However, the nucleotide mutation frequencies for subtype 1a in *NS3* and *NS5A* regions were significantly lower than those for subtype 1b (*p* = 0.002). Nucleotide mutation frequencies in the *NS5B* region as well as entropy values for all analyzed genomic regions were similar between subtype 1a and 1b (Table 6).

### 3.7. Factors Related to the Presence of Baseline RASs 

In univariate analyses, several factors were significantly associated with the presence of baseline RASs in the *NS5A*, *NS5B* and *NS3* regions (Table 7). 

In a multivariate logistic regression model, HCV subtype 1b, IFNL3 CC genotype and values above the median of Shannon entropy and nucleotide mutation frequency were identified as independent predictors of the presence of baseline RASs in the *NS5A* gene. Among these, IFNL3 CC genotype was the factor with the highest predictive value (*p* = 0.0005). In the *NS3* gene, this host genetic factor was also identified as the only independent predictor of the presence of RASs at baseline (*p* = 0.01). No factor was independently associated with the presence of RASs in the *NS5B* genomic region after the multivariate analysis (Table 7).

### 3.8. Effect of Baseline RASs on Treatment Outcome

Sixty of the 86 (69.8%) recruited patients (28 subtype 1a and 32 subtype 1b) were treated with DAAs after enrolment in this study. Most of them (43/60; 71.7%) received SOF based combination: SOF + DCV for 12 weeks (28/43, 65.1%), SOF + DCV for 24 weeks (13/43, 30.2%), SOF + SMV for 12 weeks (1/43, 2.3%), and SOF + LDV for 12 weeks (1/43, 2.3%). One patient received DCV + Peg-IFN/RBV for 24 weeks (1/60, 1.7%) and the remaining 16 patients were treated for 24 to 48 weeks during 2012 to 2013 with triple therapy including Peg-IFN/RBV in combination with a first-generation NS3 protease inhibitor: Four received BOC + Peg-IFN/RBV (4/60; 6.7%) and 12 TVR + Peg-IFN/RBV (12/60; 20%).

Overall, 93.3% (56/60) of the patients achieved SVR. Among them, 27 patients (27/56; 48.2%) exhibited at baseline a RAS (or a combination of RASs) related to the DAA-regimen. Treatment failure was observed in four (6.7%) cirrhotic and treatment-experienced patients (one subtype 1a and three subtype 1b) who relapsed after receiving triple therapy. One of the subtype 1b patients had a low-level baseline RAS (S122G) to protease inhibitors.

## 4. Discussion

The World Health Organization (WHO) has declared that HCV should be eliminated as a public health threat by 2030 [2]. Although DAAs seem a promising tool for viral elimination, HCV treatment fails in approximately 2.5% to 5% of patients and this may often occur in the presence of RASs [1]. Moreover, it has been reported that the frequencies of RASs differ between geno- and subtypes, geographical region and method of sequencing [33]. In addition, several viral, host and treatment factors can influence the emergence of RASs [18]. Therefore, in order to reach the worldwide elimination goals, RAS prevalence should be evaluated in individual countries or regions.

In this study, the combined results from both sequencing methods revealed that the frequency of natural RASs varied according to the analyzed viral region, as RASs were more frequently observed in the *NS3* when compared to their frequency in the *NS5A* (39.2% vs. 25%, respectively; *p* = 0.06) and in the *NS5B* genes (39.2% vs. 8.9%; *p* < 0.0001).

This disparity in the prevalence of RASs between the three genomic regions is a possible reflection of the fact that the substitution rates in the different proteins may be constrained by the function that each one of them fulfills in the biology of the HCV. Since the genetic variability is not evenly distributed across the viral genome [34], different evolutionary processes shape the different genetic barriers for the development of resistance of each DAA family, which are similar between NS5A and NS3 protease, but higher for NS5B polymerase [34].

In the *NS3* and *NS5A* regions, the frequencies of natural RAS were higher than those observed in previous studies [10,35], but similar to those reported in Argentina for *NS3* [25] and in Brazil [17,36,37] when taking into consideration the sequencing method and cut-off value used. Notably, when the analysis was limited to amino acid substitutions associated with a clinically relevant level of resistance (defined as “likely resistant”), their prevalence was lower in both genomic regions when compared to other studies [10,22,35,38]. In this regard, in the NS5A protein, combining the results from both sequencing methods, mutations M28V and L31M were only detected in two subtype 1a and two subtype 1b-infected patients, respectively. Moreover, RAS Y93H, which confers high-level resistance to DCV, OMV, LDV and ELB [39], was observed in one subtype 1a-infected patient at a low frequency (3%) only after performing NGS. In the *NS3* region, the clinically important RAS Q80K was only detected in one patient belonging to subtype 1a clade 1. This finding is in agreement with amino acid signature analyses from United States and Europe sequences in which Q80K polymorphism was only found in subtype 1a clade 1 and not clade 2 [40,41].

The low prevalence of the clinically relevant RASs in the *NS3* and *NS5A* regions in this study contrasts with results from Asia, Europe and USA [35,38,42,43,44], but it is similar to Argentinean and Brazilian reports [17,25,36,37]. The reasons for this discrepancy remain unclear as there is still little information about the genetic variability of the viral genes of HCV isolates from Latin America. However, this finding could be associated to geographical factors, as previously suggested [45,46].

In the *NS5B* region, the prevalence of RASs was lower than those reported by other studies [35,47]. None of the patients carried the S282T mutation, which is the signature substitution associated with resistance to SOF in vitro for all HCV genotypes [38]. This finding is in agreement with previous studies which reported that S282T is rarely (<1%) selected in patients treated with a SOF-based regimen and is not detected in treatment-naïve genotype 1 patients [42].

In this study, natural substitutions were detected at baseline in both subtypes, with a higher prevalence of RASs among subtype 1b samples in all three HCV genomic regions. This finding is in agreement with studies carried out in Italy [42], Argentina [25] and Brazil [17,35,45,48]; whereas a higher prevalence of RASs in subtype 1a sequences was observed in Europe and USA [10,22,49,50] and in two reports from Uruguay in which the number of analyzed subtype 1b sequences was relatively low [51,52].

The higher presence of Q80K among subtype 1a samples reported in Europe and USA, as well as the low prevalence of this subtype and this mutation among the samples of this study [4,25] could be possibly related to the different prevalence of RASs in each region. These geographic discrepancies highlight the genetic diversity present in both the natural RASs found in Latin American countries and the evolutionary relationships between Argentinean and other worldwide isolates, probably linked to the Italian immigration occurred in the 19th century.

Regarding the differences observed between subtypes, the nucleotide mutation frequencies in the *NS3* and *NS5A* regions revealed that the genetic diversity was significantly higher among subtype 1b-infected patients compared to those infected with subtype 1a. Subtype 1b patients were, on average, older than those infected with subtype 1a (Table 1) and may have been infected—and possibly transmitting HCV—for longer periods of time [53], leading to a higher diversity in the viral genome. Finally, the higher prevalence of multiple RASs in subtype 1b genomes could enhance their replication capacity, as was previously reported [54,55]. Taking this into account, HCV subtype 1b infection may have a more unfavorable treatment outcome in Argentina than the expected in other geographical regions, particularly with some DAA regimens. However, the baseline presence of RASs in both HCV subtypes did not appear to affect therapy outcome on a limited sample of patients treated with DAAs in this study.

The need to routinely test for RASs at baseline has been recommended for Q80K in subtype 1a prior to using SMV in interferon-containing regimens, and in patients with cirrhosis that are going to be treated with SOF and SMV. For NS5A inhibitors, it is recommended to test for RASs in subtype 1a patients when using GZR + EBR [12]. The results presented herein suggest that routine testing for RASs at baseline would not be necessary for subtype 1a patients in this population, as their frequencies are low. However, the development of a resistance monitoring system is required as this scenario may change in the near future.

It has been reported that not only the presence of a particular RAS but also the RAS dominancy (>15% to 25%) in the patient’s quasispecies has an impact on treatment outcome [56,57], although a recent study suggests that minority RASs present at much lower than 15% may also be relevant for treatment planning [58]. In this regard, this is the first report in Argentina that screened HCV drug resistance using NGS. This technology revealed RASs—not detected by automated sequencing—in 10.5% and 11.1% of the *NS5A* and *NS3* samples. Among them, only two RASs were detected at a frequency above 15% among the patient’s viral quasispecies: M28V (with a frequency of 21%) in *NS5A* and Q80L (with a frequency of 21%) in *NS3*. This finding supports the fact that deep sequencing has not been proven clinically relevant for RASs testing [59]. Indeed, NGS methodology is costly, too laborious, and technically challenging for many laboratories [60] and, thus, NGS could not yet be ready for routine clinical practice in Argentina.

Although Sanger automated sequencing is generally limited to the detection of variants with greater than 20% of prevalence [16], the two RASs mentioned above could not be detected by automated sequencing. This lack of correlation between both sequencing methods was probably due to the fact that both samples exhibited low viral loads (3.2 log_10_ copies/mL), as it was previously reported [61]. In fact, it has been estimated that the efficiency of RNA extraction and cDNA synthesis is 1% to 10% [62]; thus, in low viral load samples, the small number of molecules present in the cDNA could be better examined by the higher analytical sensitivity of NGS [63]. 

Regarding host factors, it has been reported that the IFNL4 TT genotype, which has a more efficient antiviral response to HCV infection, is strongly associated with RAS Y93H in the subtype 1b NS5A protein, resulting in selection for viral adaptations [19,21]. In this study, univariate and multivariate analyses revealed that the IFNL3 CC genotype was identified as an independent predictor of the presence of RASs at baseline in *NS5A* and *NS3* genomic regions. Therefore, differences in the frequency of *NS5A* and *NS3* RASs could reflect disparities in the selection pressure against HCV due to genetic variations within the IFNL3/4 locus. 

Finally, this study has certain limitations that need to be considered. First, *NS3*, *NS5A* and *NS5B* genes could not be amplified in some patients due to the low volume of stored serum and/or RNA samples. Second, the number of analyzed sequences was not so large. However, other studies included a similar—or even smaller—number of patients [46,51,52]. Finally, this study recruited only genotype 1 patients from Buenos Aires and the metropolitan region, and thus excluded patients infected with other genotypes or from distant areas of Buenos Aires, where the ancestry composition of the population and the prevalence of host genetic factors differ [64]. Therefore, these findings should not be generalized or transferable to the whole country.

## 5. Conclusions

Decisions regarding which combinations of DAAs to use remain challenging [65,66]. This study depicts a current scenario on DAA resistance in Buenos Aires, Argentina. In summary, the frequencies of natural *NS5A* and *NS3* substitutions were similar than those previously reported in this geographic region. In the *NS5B* region, the prevalence of RASs was lower than those previously reported. Notably, the prevalence of clinically relevant RASs in the three analyzed genes was lower than those observed around the world. Although tested on a limited sample of patients in this study, the baseline presence of RASs in both HCV subtypes did not appear to affect therapy outcome. Despite the fact that NGS allowed a more complete analysis of RASs, its usefulness may be limited in this population. These results support the need to define the resistance testing in each country since the RASs have very different worldwide prevalence.

## Figures and Tables

**Table 1:** Main characteristics of the Hepatitis C Virus (HCV) genotype 1 patients recruited in this study.

Characteristics	All (*n* = 86)	HCV Subtype		*p* Value
		HCV GT1a (*n* = 33, 38.4%)	HCV GT1b (*n* = 53, 61.6%)	
HCV viral load, log_10_ copies/mL, mean ± SD	6.1 ± 3.8	6.2 ± 3.9	6.1 ± 3.5	0.71
Age, years, mean ± SD	54.7 ± 11.0	50.6 ± 8.9	57.3 ± 11.5	**0.005**
Male, no. (%)	52 (60.5)	24 (72.7)	28 (52.8)	0.07
METAVIR Score, no. (%)				
F0/F1	38 (44.2)	11 (33.3)	27 (50.9)	
F2	18 (20.9)	10 (30.3)	8 (15.1)	0.1
F3	10 (11.6)	6 (18.2)	4 (7.5)	
F4	20 (23.3)	6 (18.2)	14 (26.5)	
HIV co-infection, no. (%)	5 (5.8)	3 (9.1)	2 (3.7)	0.37
Previous failure to PegIFN/RBV treatment, no. (%)	31 (36.1)	12 (36.4)	19 (35.9)	1
IFNL3 SNP rs12979860, no. (%)				
CC	18 (20.9)	9 (27.3)	9 (17)	
CT	57 (66.3)	19 (57.6)	38 (71.7)	0.39
TT	11 (12.8)	5 (15.1)	6 (11.3)	
IFNL4 SNP rs368234815, no. (%)				
TT/TT	18 (20.9)	9 (27.3)	9 (17)	
TT/ΔG	57 (66.3)	19 (57.6)	38 (71.7)	0.39
ΔG/ΔG	11 (12.8)	5 (15.1)	6 (11.3)	

Statistically significant *p* values are in bold.

**Table 2:** Frequency of resistance-associated substitutions (RASs) in the NS5A protein by automated Sanger sequencing data.

Genotype	Reference NS5A Position	RASs	Frequency
	K24	R	-
	M28	A/G/T/S/V	M28V (1/33; 3%)
	Q30	D/E/G/H/K/L/N/R/Y	-
	L31	F/I/M/V	-
	P32	L	-
1a	S38	F	-
	H58	D	-
	A92	K/T	-
	Y93	C/F/H/L/N/R/S/T/W	-
	Q24	K	-
	L28	M/T	L28M (1/51; 2%)
	R30	G/H/Q/S	R30Q (7/51; 13.7%)
1b	L31	F/I/M/V	L31M (1/51; 2%) L31F (1/51; 2%)
	P58	A/D/L/R/S/T	P58R (1/51; 2%) P58T (1/51; 2%)
	A92	K/T	A92T (1/51; 2%)
	Y93	C/H/I/N/R/S/T	-

**Table 3:** Frequency of RASs in the NS5B polymerase by automated Sanger sequencing data.

Genotype	Reference NS5B Position	RASs	Frequency
	L159	F	-
1a	E237	G	-
	S282	R/T	-
1b	L159	F	L159F (5/49; 10.2%)
	S282	G/T	S282G (2/49; 4.1%)

**Table 4:** Frequency of RASs in the NS3 protease by automated Sanger sequencing data.

Genotype	Reference NS3 Position	RASs	Frequency
	V36	A/G/M/L	V36L (1/29; 3.4%)
	Q41	R	-
	F43	S	-
	T54	A/S	T54S (1/29; 3.4%)
	Y56	F/H	-
1a	Q80	K/R	Q80K (1/29; 3.4%)
	S122	R	-
	R155	I/G/K/N/Q/S/T/W	-
	A156	G/L/M/S/T/V	-
	V158	A	-
	D168	A/C/E/F/G/H/I/K/L/N/R/S/T/V/Y	-
	I170	T/V	I170V (1/29; 3.4%)
	V36	A/G/M	-
	Q41	R	-
	F43	L/S/V	-
	T54	A/S	T54S (1/50; 2%)
	V55	A/I	-
	Y56	F/H	Y56F (8/50; 16%)
1b	Q80	K/L/R	Q80K (1/50; 2%) Q80R (1/50; 2%)
	S122	D/G/R/T	S122G (4/50; 8%) S122T (2/50; 4%)
	R155	K/G/L/T/Q/W	R155K (1/50; 2%)
	A156	G/S/T/V	-
	D168	A/C/E/F/G/H/I/K/N/Q/T/V/Y	-
	V170	A/T	-
	M175	L	-

**Table 5:** Prevalence of RASs in the *NS5A* and *NS3* regions detected only by Next Generation Sequencing.

Genomic Region	RASs	Patient No.	HCV Subtype	Mutation Frequency in Viral Quasispecies
	K24R	87	1a	K24R (2%)
	Q24K	80	1b	Q24K (6.7%)
	M28A/G/T/S/V	4	1a	M28V (21%)
NS5A	R30G/H/Q/S	10	1b	R30H (5.8%)
	L31F/I/M/V	24	1b	L31M (5.3%)
	P58A/D/L/R/S/T	97	1b	P58S (3.4%)
	A92K/T	120	1b	A92T (4.2%)
	Y93C/F/H/L/N/R/S/T/W	4	1a	Y93H (3%)
	F43L/S/V	108	1b	F43S (2%)
	T54A/S	62	1b	T54S (3.5%)
	Y56F/H	106	1b	Y56F (3.9%)
		90	1b	Y56F (7.3%)
NS3		74	1b	Y56H (4.1%)
	Q80K/L/R	40	1b	Q80L (21%)
	S122D/G/R/T	49	1b	S122G (4.8%)
		88	1b	S122G (12%)
		76	1b	S122T (3.9%)

**Table 6:** Mean genetic complexity in each viral genomic region by HCV subtype.

Genomic Regions	HCV Subtype (*n*=)		Mutations ^1^/Nucleotides Sequenced		Nucleotide Mutation Frequency ^2^		*p* Value	Normalized Shannon Entropy ^3^		*p* Value
	1a	1b	1a	1b	1a	1b		1a	1b	
*NS5A*	32	44	50/711	75/715	0.07	0.1	**0.002**	0.011	0.017	0.11
*NS5B*	30	42	73/916	78/945	0.08	0.08	0.86	0.014	0.017	0.85
*NS3*	29	43	74/1007	132/1300	0.07	0.1	**0.002**	0.015	0.017	0.19

^1^ Mutations are those that vary when compared to the corresponding reference sequences; ^2^ the nucleotide mutation frequency is the total number of mutations divided by the total number of nucleotides sequenced; ^3^ the normalized Shannon entropy is calculated as Sn = −[∑i (pi ln pi)]/ln N, in which pi is the proportion of each sequence of the mutant spectrum and N is the total number of sequences compared. Statistically significant *p* values are in bold.

**Table:** **A**

Patients Characteristics	RASs (*n* = 14)	No RASs (*n* = 70)	Univariate Analysis	Multivariate Analysis	
			*p* Value	*p* Value	OR (95% CI)
Male gender, no. (%)	9 (64.3)	41 (58.6)	0.77		
Age (years), mean ± SD	53.4 ± 5.1	56.5 ± 4.9	0.77		
HCV viral load (log_10_), mean ± SD	6.2 ± 3.2	6.1 ± 3.3	0.47		
HCV subtype 1b, no. (%)	13 (92.9)	38 (54.3)	**0.007**	**0.03**	**16.2 (1.3–20.6)**
METAVIR Score F4, no. (%)	6 (42.9)	13 (18.6)	0.07		
HIV co-infection, no. (%)	2 (14.3)	3 (4.3)	0.19		
Previous failure to PegIFN/RBV treatment, no. (%)	8 (57.1)	23 (32.9)	0.13		
IFNL3 SNP (rs12979860) CC genotype ^a^, no. (%)	10 (71.4)	8 (11.4)	**0.0001**	**0.0005**	**16.8 (3.4–8.2)**
Normalized Shannon Entropy ^b^, mean ± SD (×1000)	17 ± 8.7	10.3 ± 3.6	**0.02**	**0.01**	**2.16 (1.3–4.6)**
Nucleotide Mutation Frequency ^b^, mean ± SD (×1000)	9.8 ± 4.5	6.3 ± 2.1	**0.004**	**0.009**	**4.7 (1.4–15.2)**

Statistically significant *p* values are in bold. OR: Odds Ratio; 95% CI: 95% confidence interval. ^a^ IFNL4 (rs368234815) was not included in the analysis due to its high linkage disequilibrium with IFNL3 (rs12979860) SNP in the analyzed population (Table 1). ^b^ Calculated on 76 samples.

**Table:** **B**

Patients Characteristics	RASs (*n* = 7)	No RASs (*n* = 72)	Univariate Analysis	Multivariate Analysis	
			*p* Value	*p* Value	OR (95% CI)
Male gender, no. (%)	5 (71.4)	43 (59.7)	0.7		
Age (years), mean ± SD	53.5 ± 5.4	50.3 ± 5.6	0.52		
HCV viral load (log_10_), mean ± SD	6.1 ± 2.2	5.9 ± 2.6	0.88		
HCV subtype 1b, no. (%)	7 (100)	42 (58.3)	**0.04**	0.99	1.7 (0.40–1.41)
METAVIR Score F4, no. (%)	3 (42.9)	17 (23.6)	0.36		
HIV co-infection, no. (%)	1 (14.3)	4 (5.55)	0.38		
Previous failure to PegIFN/RBV treatment, no. (%)	5 (71.4)	29 (40.3)	0.13		
IFNL3 SNP (rs12979860) CC genotype ^a^, no. (%)	2 (28.6)	12 (16.7)	0.6		
Normalized Shannon Entropy ^b^, mean ± SD (×1000)	8.9 ± 2.3	9.6 ± 1.4	0.4		
Nucleotide Mutation Frequency ^b^, mean ± SD (×1000)	6.2 ± 3.9	2.9 ± 3.1	**0.03**	0.98	1 (0.78–1.3)

Statistically significant *p* values are in bold. OR: Odds Ratio; 95% CI: 95% confidence interval. ^a^ IFNL4 (rs368234815) was not included in the analysis due to its high linkage disequilibrium with IFNL3 (rs12979860) SNP in the analyzed population (Table 1). ^b^ Calculated on 72 samples.

**Table:** **C**

Patients Characteristics	RASs (*n* = 22)	No RASs (*n* = 57)	Univariate Analysis	Multivariate Analysis	
			*p* Value	*p* Value	OR (95% CI)
Male gender, no. (%)	17 (77.2)	36 (63.2)	0.29		
Age (years), mean ± SD	55.1 ± 4.8	52.5 ± 6.1	0.59		
HCV viral load (log_10_), mean ± SD	6.2 ± 3.1	5.9 ± 1.9	0.27		
HCV subtype 1b, no. (%)	18 (81.8)	32 (56.1)	**0.04**	0.57	1.51 (0.36–6.4)
METAVIR Score F4, no. (%)	8 (36.4)	12 (21.05)	0.25		
HIV co-infection, no. (%)	2 (9.1)	3 (5.3)	0.61		
Previous failure to PegIFN/RBV treatment, no. (%)	10 (45.4)	16 (28.1)	0.18		
IFNL3 SNP (rs12979860) CC genotype ^a^, no. (%)	12 (54.5)	10 (17.5)	**0.002**	**0.01**	**6.3 (1.5–25.8)**
Normalized Shannon Entropy ^b^, mean ± SD (×1000)	21.2 ± 1.5	15.8 ± 2.9	0.33		
Nucleotide Mutation Frequency ^b^, mean ± SD (×1000)	7.9 ± 2.6	6.8 ± 4.1	0.21		

Statistically significant *p* values are in bold. OR: Odds Ratio; 95% CI: 95% confidence interval. ^a^ IFNL4 (rs368234815) was not included in the analysis due to its high linkage disequilibrium with IFNL3 (rs12979860) SNP in the analyzed population (Table 1). ^b^ Calculated on 72 samples.

## Supplementary Materials

The following are available online at http://www.mdpi.com/1999-4915/11/1/3/s1, Figure S1: Maximum-likelihood phylogenetic tree (GTR+Γ+I model for nucleotide substitutions) including 48 references sequences (in black) and the 84 HCV-*NS5A* samples of this study (in red). The numbers at each node correspond to bootstrap values obtained with 100 replicates (values lower than 70 are not shown); Figure S2: Maximum-likelihood phylogenetic tree (GTR+Γ+I model for nucleotide substitutions) including 48 references sequences (in black) and the 79 HCV-*NS5B* samples of this study (in red). The numbers at each node correspond to bootstrap values obtained with 100 replicates (values lower than 70 are not shown); Figure S3: Maximum-likelihood phylogenetic tree (GTR+Γ+I model for nucleotide substitutions) including 48 references sequences (in black) and the 79 HCV-*NS3* samples of this study (in red). The numbers at each node correspond to bootstrap values obtained with 100 replicates (values lower than 70 are not shown).
