# Supplementary material for: Prevalence and Factors Related to Natural Resistance-Associated Substitutions to Direct-Acting Antivirals in Patients with Genotype 1 Hepatitis C Virus Infection
Source: Viruses. 2018 Dec 21;11(1):3. doi: 10.3390/v11010003 (PMC6356817; doi:10.3390/v11010003)
Supplement: Supplementary file 1 [file viruses-11-00003-s001.pdf]

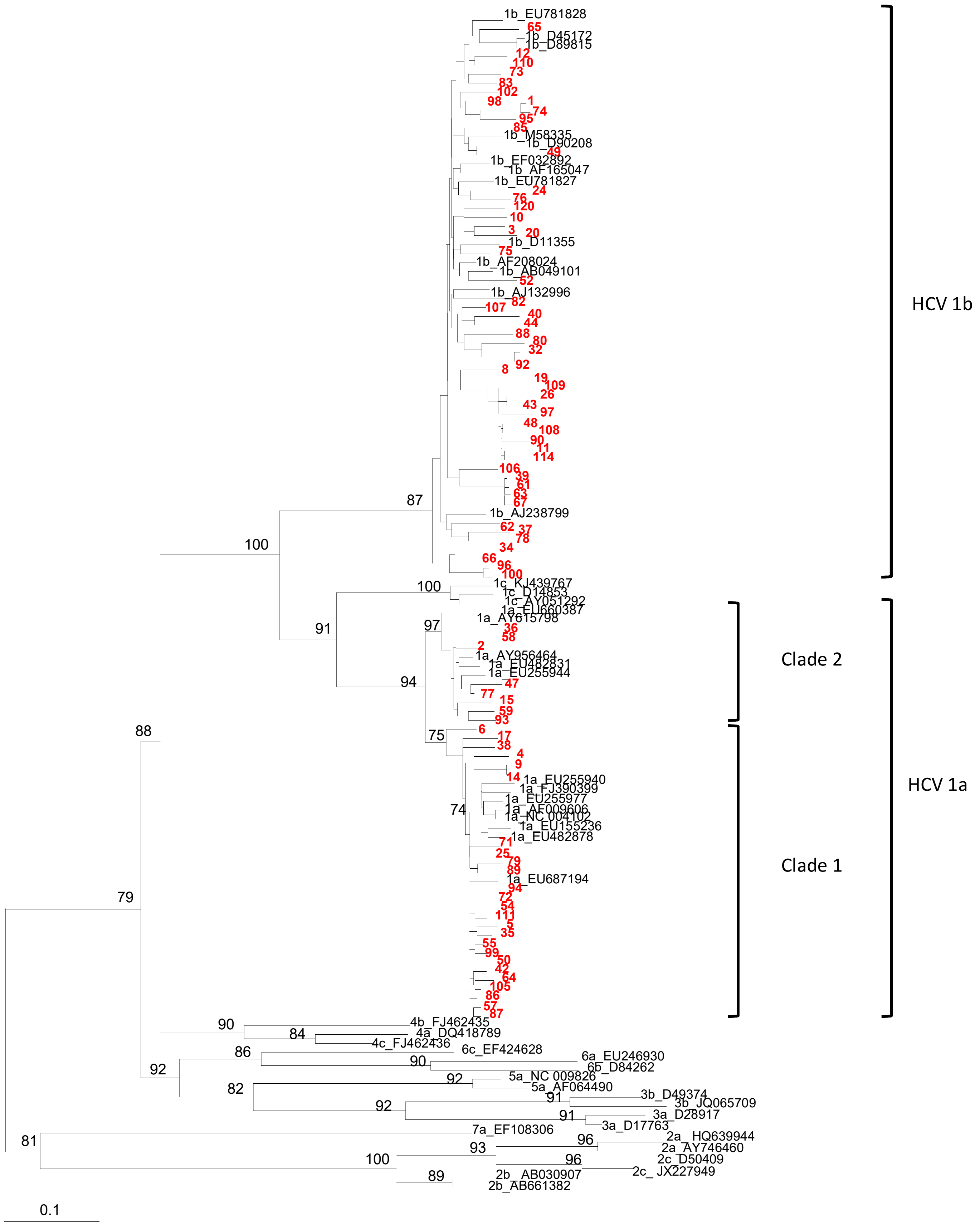

HCV 1b
HCV 1a
Clade 2
Clade 1
1b_EU781828
1b_D45172
1b_D89815
1b_M58335
1b_D90208
1b_EF032892
1b_AF165047
1b_EU781827
1b_D11355
1b_AF208024
1b_AB049101
1b_AJ132996
1b_AJ238799
1c_KJ439767
1c_D14853
1c_AY051292
1a_EU660387
1a_AY615798
1a_AY956464
1a_EU482831
1a_EU255944
1a_EU255940
1a_FJ390399
1a_EU255977
1a_AF009606
1a_NC_004102
1a_EU155236
1a_EU482878
1a_EU687194
4b_FJ462435
4a_DQ418789
4c_FJ462436
6c_EF424628
6a_EU246930
6b_D84262
5a_NC_009826
5a_AF064490
3b_D49374
3b_JQ065709
3a_D28917
3a_D17763
7a_EF108306
2a_HQ639944
2a_AY746460
2c_D50409
2c_JX227949
2b_AB030907
2b_AB661382
0.1

Supplementary Figure S1. Maximum-likelihood phylogenetic tree (GTR+Γ+I model for nucleotide substitutions) including 48 references sequences (in black) and the 84 HCV-*NS5A* samples of this study (in red). The numbers at each node correspond to bootstrap values obtained with 100 replicates (values lower than 70 are not shown).

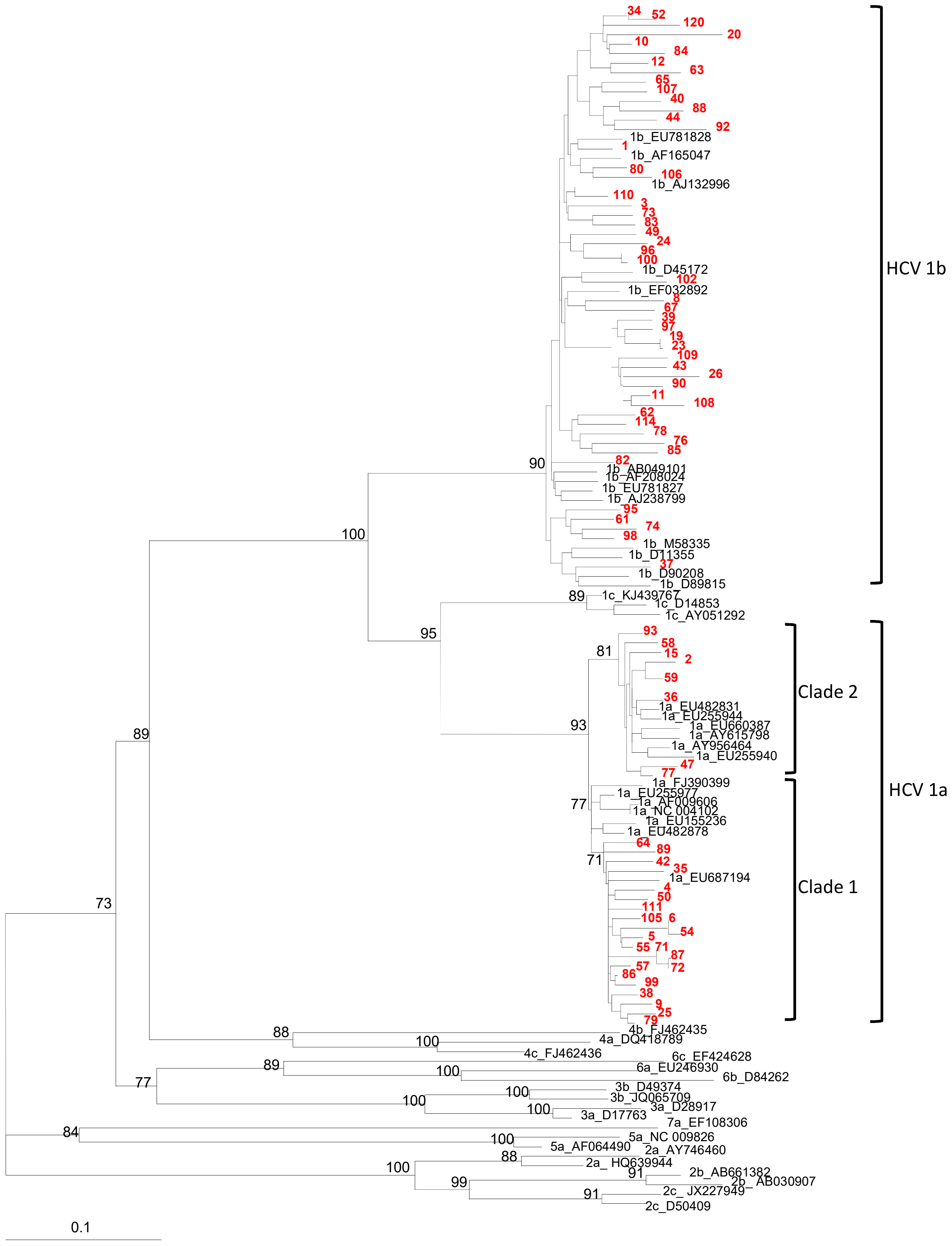

34
52
120
20
10
84
12
63
65
107
40
88
44
92
1b_EU781828
1
1b_AF165047
80
106
1b_AJ132996
110
3
73
83
49
24
96
100
1b_D45172
102
1b_EF032892
8
67
39
97
19
23
109
43
26
90
11
108
62
114
78
76
85
82
90
1b_AB049101
1b_AF208024
1b_EU781827
1b_AJ238799
95
61
74
98
100
1b_M58335
1b_D11355
1b_D90208
37
1b_D89815
89
1c_KJ439767
1c_D14853
1c_AY051292
95
93
58
15
2
59
81
36
1a_EU482831
1a_EU255944
93
1a_EU660387
1a_AY615798
1a_AY956464
1a_EU255940
47
77
1a_FJ390399
1a_EU255977
77
1a_AF009606
1a_NC 004102
1a_EU155236
1a_EU482878
64
89
71
42
35
1a_EU687194
4
50
111
105
6
54
5
55
71
87
72
57
86
99
38
9
25
79
4b_FJ462435
88
4a_DQ418789
100
4c_FJ462436
89
6c_EF424628
6a_EU246930
100
6b_D84262
77
100
3b_D49374
3b_JQ065709
100
3a_D28917
100
3a_D17763
73
89
7a_EF108306
84
100
5a_NC 009826
5a_AF064490
2a_AY746460
88
2a_HQ639944
100
91
2b_AB661382
2b_AB030907
99
2c_JX227949
91
2c_D50409
0.1
HCV 1b
Clade 2
HCV 1a
Clade 1

Supplementary Figure S2. Maximum-likelihood phylogenetic tree (GTR+Γ+I model for nucleotide substitutions) including 48 references sequences (in black) and the 79 HCV-*NS5B* samples of this study (in red). The numbers at each node correspond to bootstrap values obtained with 100 replicates (values lower than 70 are not shown).

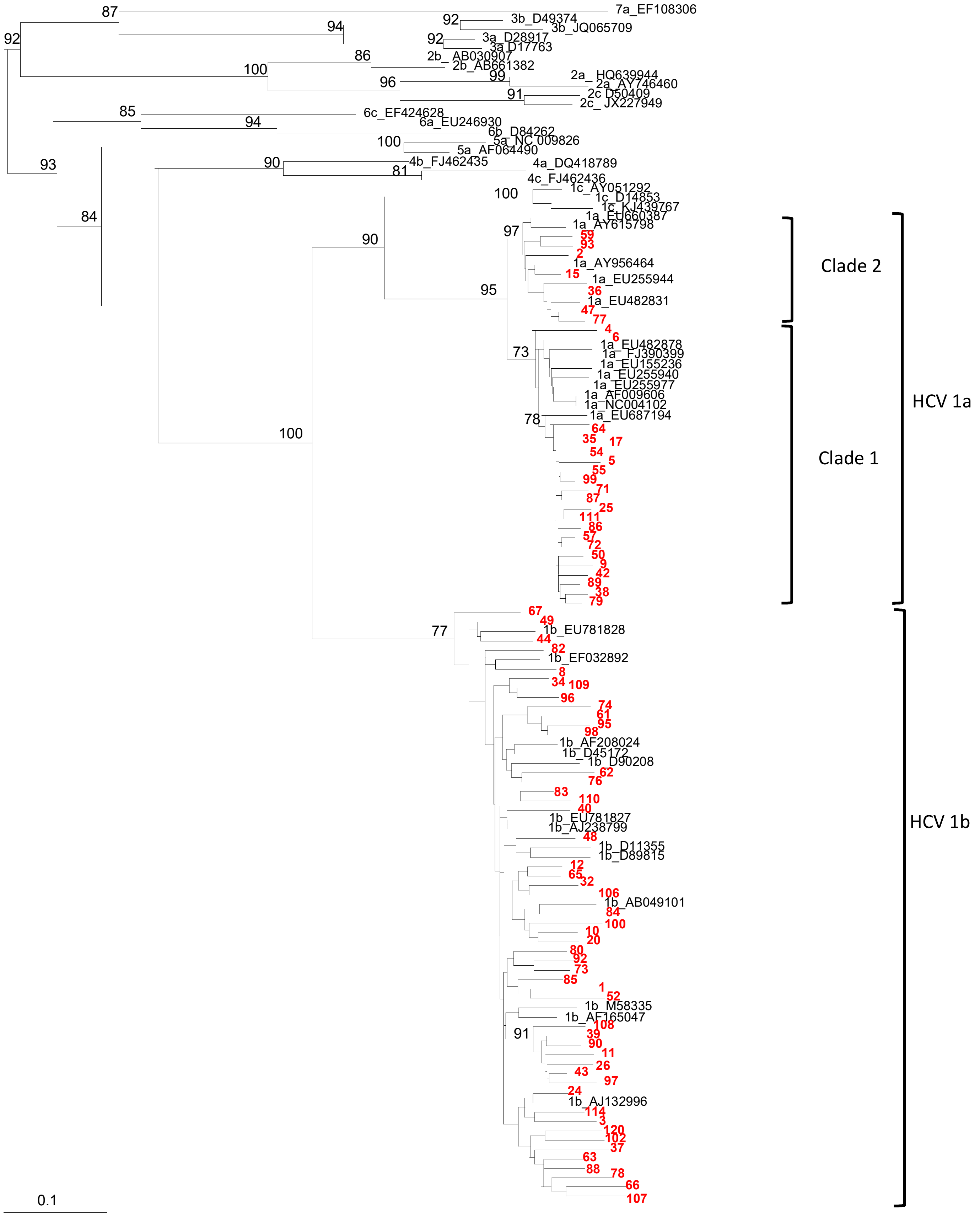

7a_EF108306
3b_D49374
3b_JQ065709
3a_D28917
3a_D17763
2b_AB030907
2b_AB661382
2a_HQ639944
2a_AY746460
2c_D50409
2c_JX227949
6c_EF424628
6a_EU246930
6b_D84262
5a_NC_009826
5a_AF064490
4b_FJ462435
4a_DQ418789
4c_FJ462436
1c_AY051292
1c_D14853
1c_KJ439767
1a_EU660387
1a_AY615798
1a_AY956464
1a_EU255944
1a_EU482831
1a_EU482878
1a_FJ390399
1a_EU155236
1a_EU255940
1a_EU255977
1a_AF009606
1a_NC004102
1a_EU687194
1b_EU781828
1b_EF032892
1b_AF208024
1b_D45172
1b_D90208
1b_EU781827
1b_AJ238799
1b_D11355
1b_D89815
1b_AB049101
1b_M58335
1b_AF165047
1b_AJ132996
Clade 2
Clade 1
HCV 1a
HCV 1b
0.1

Supplementary Figure S3. Maximum-likelihood phylogenetic tree (GTR+Γ+I model for nucleotide substitutions) including 48 references sequences (in black) and the 79 HCV-*NS3* samples of this study (in red). The numbers at each node correspond to bootstrap values obtained with 100 replicates (values lower than 70 are not shown).
